# The maize cytochrome P450 CYP79A61 produces phenylacetaldoxime and indole-3-acetaldoxime in heterologous systems and might contribute to plant defense and auxin formation

**DOI:** 10.1186/s12870-015-0526-1

**Published:** 2015-05-29

**Authors:** Sandra Irmisch, Philipp Zeltner, Vinzenz Handrick, Jonathan Gershenzon, Tobias G. Köllner

**Affiliations:** Department of Biochemistry, Max Planck Institute for Chemical Ecology, Hans-Knöll Straße 8, 07745 Jena, Germany

**Keywords:** Maize, P450, CYP79, Herbivory, Aldoxime, Auxin, Cyanogenic glycoside

## Abstract

**Background:**

Plants produce a group of aldoxime metabolites that are well known as volatiles and as intermediates in cyanogenic glycoside and glucosinolate biosynthesis in particular plant families. Recently it has been demonstrated that aldoximes can also accumulate as part of direct plant defense in poplar. Cytochrome P450 enzymes of the CYP79 family were shown to be responsible for the formation of aldoximes from their amino acid precursors.

**Results:**

Here we describe the identification and characterization of maize CYP79A61 which was heterologously expressed in yeast and *Nicotiana benthamiana* and shown to catalyze the formation of (*E/Z*)-phenylacetaldoxime and (*E/Z*)-indole-3-acetaldoxime from L-phenylalanine and L-tryptophan, respectively. Simulated herbivory on maize leaves resulted in an increased *CYP79A61* transcript accumulation and in elevated levels of L-phenylalanine and (*E/Z*)-phenylacetaldoxime. Although L-tryptophan levels were also increased after the treatment, (*E/Z*)-indole-3-acetaldoxime could not be detected in the damaged leaves. However, simulated herbivory caused a significant increase in auxin concentration.

**Conclusions:**

Our data suggest that CYP79A61 might contribute to the formation of (*E/Z*)-phenylacetaldoxime in maize. Since aldoximes have been described as toxic compounds for insect herbivores and pathogens, the increased accumulation of (*E/Z*)-phenylacetaldoxime after simulated herbivory indicates that this compound plays a role in plant defense. In addition, it is conceivable that (*E/Z*)-indole-3-acetaldoxime produced by recombinant CYP79A61 could be further converted into the plant hormone indole-3-acetic acid after herbivore feeding in maize.

**Electronic supplementary material:**

The online version of this article (doi:10.1186/s12870-015-0526-1) contains supplementary material, which is available to authorized users.

## Background

Aldoximes, a group of nitrogen-containing plant secondary metabolites, have been intensively studied as key intermediates in the biosynthesis of plant defense compounds such as glucosinolates, cyanogenic glycosides, and various phytoalexins [1–3]. Moreover, these compounds are known to be released as volatiles from flowers and vegetative organs of a multitude of plant species [4]. In general, aldoximes are produced from their corresponding amino acid precursors through the action of cytochrome P450 monooxygenases (CYPs) of the CYP79 family (recently reviewed in [5]). Members of this family have been identified from several plant species and the presence of putative *CYP79* genes in all angiosperm genomes sequenced so far suggests a widespread distribution of CYP79s in higher plants [6]. The first reported CYP79 enzyme, CYP79A1, was isolated from sorghum (*Sorghum bicolor*) and catalyzes the conversion of L-tyrosine to *p*-hydroxyphenylacetaldoxime which is the precursor of dhurrin, the major cyanogenic glycoside in sorghum [7]. CYP79B2 and CYP79B3 from Arabidopsis are two examples of CYP79 enzymes involved in glucosinolate and phytoalexin formation. Both enzymes accept L-tryptophan as substrate and produce indole-3-acetaldoxime which is further converted into indole glucosinolates and camalexin in Arabidopsis [8, 9]. The aldoxime intermediates produced by CYP79 enzymes do not accumulate in the plant but are channeled within a large protein complex called a metabolon [10].

Recently, it has been shown that CYP79 enzymes are also responsible for the production of volatile aldoximes. The two enzymes CYP79D6v3 and CYP79D7v2 from *Populus trichocarpa* catalyze the formation of (*E/Z*)-2-methylbutyraldoxime, (*E/Z*)-3-methylbutyraldoxime, and (*E/Z*)-isobutyraldoxime from L-isoleucine, L-leucine, and L-valine, respectively [6]. The aldoximes produced are characteristic components of the herbivore-induced volatile blend of poplar and it has been demonstrated that they are involved in the attraction of natural enemies of herbivores [11]. In addition to the volatile aliphatic aldoximes which are released from poplar without detectable accumulation in the plant, CYP79D6v3 and CYP79D7v2 also produce the less volatile (*E/Z*)-phenylacetaldoxime. This compound was found to accumulate in poplar leaves after herbivore feeding and bioassays using pure (*E/Z*)-phenylacetaldoxime revealed a toxic effect against a generalist lepidopteran herbivore, suggesting that aldoxime accumulation may contribute to direct plant defense against insects [6].

During the last two decades, maize (*Zea mays*) has become an important model species for studying plant-insect interactions on a physiological and molecular level. As many other plants, maize responds to caterpillar feeding by the expression of a complex arsenal of defense reactions such as the accumulation of secondary compounds [12, 13], the formation of defensive proteins [14, 15], and the release of volatiles [16]. Despite the intensive research on maize, there is little information about the occurrence of aldoximes and aldoxime-derived defense compounds in this plant species. A few early papers reported maize as a cyanogenic species. However, the measured hydrogen cyanide content was rather low in comparison to sorghum and other cyanogenic plants, and a cyanogenic glycoside could not be identified in maize so far [17–19]. The emission of aliphatic aldoximes from herbivore-damaged maize has been reported for two different cultivars [20, 21] but it seems that the majority of maize germplasm is not able to generate such compounds [22, 23]. However, a recent survey of all available plant genomes revealed the presence of four putative *CYP79* genes in the maize genome [6]. We have now begun to study these enzymes and their contribution to aldoxime production in maize.

This paper reports the characterization of CYP79A61, an enzyme able to convert L-phenylalanine and L-tryptophan into phenylacetaldoxime and indole-3-acetaldoxime, respectively. Simulated herbivory on maize leaves resulted in the upregulation of *CYP79A61* gene expression and in an increase in amino acid substrate accumulation, corresponding to higher levels of phenylacetaldoxime in treated plants in comparison to undamaged control plants. Since indole-3-acetic acid (IAA) was also significantly upregulated after the treatment, we propose that CYP79A61 plays a role in herbivore-induced auxin formation.

## Results

### Maize possesses four *CYP79* genes

**Fig. 1:**
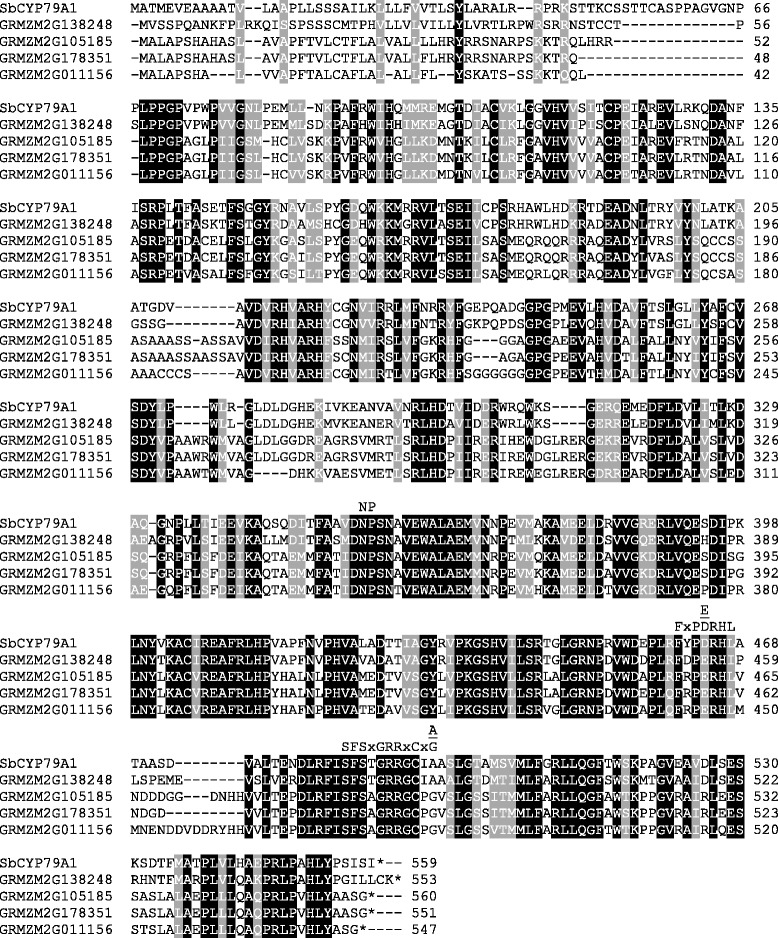
Comparison of the amino acid sequences of putative maize CYP79s with sorghum CYP79A1. Amino acids identical in all five sequences are marked by black boxes and amino acids with similar side chains are marked by gray boxes. Sequence motifs characteristic for CYP79 proteins are labeled

**Fig. 2:**
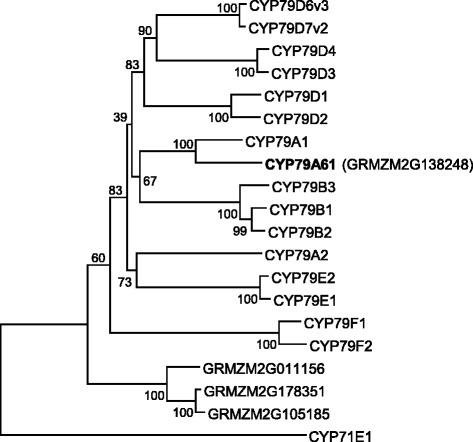
Phylogenetic tree of CYP79 sequences from maize and previously characterized CYP79 enzymes from other plant species. The rooted tree was inferred with the neighbor-joining method and *n* = 1000 replicates for bootstrapping. Bootstrap values are shown next to each node. As an outgroup, CYP71E1 from *Sorghum bicolor* was chosen. Accession numbers are given in the Methods section

In a previous study on poplar CYP79 enzymes [6], we performed a BLAST analysis with all available angiosperm genomes to study the distribution of *CYP79* genes in higher plants. Among others this analysis revealed the presence of four putative *CYP79* sequences in the genome of the maize inbred line B73. The open reading frames of the four genes GRMZM2G138248, GRMZM2G011156, GRMZM2G105185, and GRMZM2G178351 encode for proteins with 552, 546, 559, and 550 amino acids, respectively (Fig. 1). Motifs reported to be conserved in CYP79 proteins such as the heme binding site (SFSxGRRxCxA/G), the PERH motif, and the NP motif in one of the substrate binding sites were also found in the identified maize CYP79 sequences (Fig. 1). A phylogenetic analysis using these sequences and already characterized CYP79s from other plant species showed that GRMZM2G138248 clustered together with sorghum CYP79A1 (72 % amino acid identity) while the other three maize proteins GRMZM2G011156, GRMZM2G105185, and GRMZM2G178351 formed a separate clade in the basal part of the phylogenetic tree (Fig. 2). A synteny analysis of the maize and sorghum genomes revealed that GRMZM2G138248 and sorghum *CYP79A1* seem not to represent orthologous genes since they were found to be located in non-syntenic genomic regions (Additional file 1: Figure S1). However, the putative sorghum *CYP79* gene Sb10g022470 which encodes a protein with 83.3 % amino acid sequence similarity to GRMZM2G138248 could be identified as a likely orthologue of GRMZM2G138248 (Additional file 1: Figures S2 and S3).

We tried to amplify the maize *CYP79* genes from cDNA made from herbivore-damaged seedlings of the commercial hybrid line Delprim, a cultivar commonly used in maize-insect interaction studies. While the complete open reading frame of GRMZM2G138248 could be isolated from the cDNA, the amplification of GRMZM2G011156, GRMZM2G105185, and GRMZM2G178351 failed, suggesting that these genes were not present in Delprim or not expressed in seedlings under the experimental conditions. The GRMZM2G138248 gene obtained was designated *CYP79A61* following the standard P450 nomenclature (D.R. Nelson, P450 Nomenclature Committee).

### CYP79A61 produces *(E)*- and *(Z)*-isomers of phenylacetaldoxime and indole-3-acetaldoxime after yeast expression

**Fig. 3:**
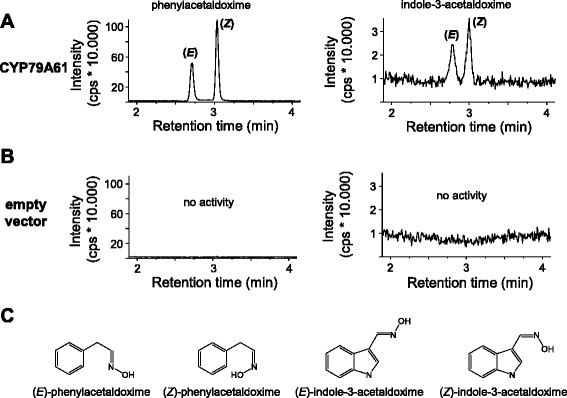
Catalytic activity of CYP79A61. Yeast microsomes containing the heterologously-expressed enzyme **a** or an empty vector control **b** were prepared and incubated with the potential substrates L-phenylalanine and L-tryptophan. Products were detected using LC-MS/MS analysis with multiple reaction monitoring in the positive mode. Diagnostic reactions for each product: phenylacetaldoxime, *m/z* 136.0/119.0; indole-3-acetaldoxime, *m/z* 175.0/158.0. The structures of all detected CYP79A61 products are shown in **c**

**Fig. 4:**
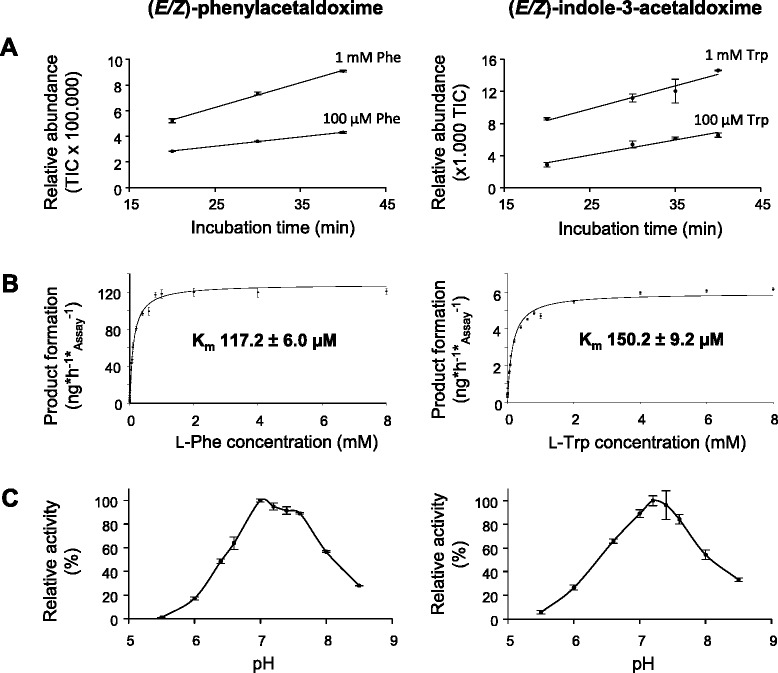
Biochemical characterization of CYP79A61. Yeast microsomes containing the heterologously-expressed enzyme were prepared and incubated with the substrates L-phenylalanine and L-tryptophan. Time courses for the product formation in the presence of either 100 μM or 1 mM substrate are shown in **a**. The Michaelis-Menten kinetics for L-phenylalanine and L-tryptophan are given in **b** and the pH dependency of CYP79A61 product formation is illustrated in **c**. Products were detected using LC-MS/MS analysis with multiple reaction monitoring in the positive mode. Diagnostic reactions for each product: phenylacetaldoxime, *m/z* 136.0/119.0; indole-3-acetaldoxime, *m/z* 175.0/158.0

For heterologous expression in yeast (*Saccharomyces cerevisiae*), the complete open reading frame of *CYP79A61* was cloned into the vector pESC-Leu2d [24] and the resulting construct was transferred into the *S. cerevisiae* strain WAT11 which carries the Arabidopsis cytochrome P450 reductase 1 (CPR1) [25]. Prepared microsomes containing recombinant CYP79A61 and CPR1 were incubated with the potential amino acid substrates L-phenylalanine, L-tyrosine, L-tryptophan, L-isoleucine, and L-leucine in the presence of the electron donor NADPH. Enzyme products were detected using liquid chromatography-tandem mass spectrometry (LC-MS/MS) analysis and verified by the use of authentic standards prepared as described in the Methods section. CYP79A61 accepted L-phenylalanine and L-tryptophan as substrates and converted them into mixtures of the (*E*)- and (*Z*)-isomers of phenylacetaldoxime and indole-3-acetaldoxime, respectively (Fig. 3). No activity could be observed with L-tyrosine, L-isoleucine, and L-leucine. The pH optima for the formation of phenylacetaldoxime and indole-3-acetaldoxime were 7.0 and 7.2, respectively, and the substrate affinity for L-phenylalanine (*K*_m_ = 117.2 ± 6.0 μM) was slightly higher than that for L-tryptophan (*K*_m_ = 150.2 ± 9.2 μM) (Fig. 4). Since measurements of carbon monoxide difference spectra were inconclusive, we were not able to determine the protein concentrations in the microsomes and thus to calculate the turnover numbers for the different substrates. However, the large difference between the maximal velocities (*V*_max_) for 1 mM L-phenylalanine (118.3 ± 3.7 ng (*E/Z*)-phenylacetaldoxime*h^−1^*assay^−1^) and 1 mM L-tryptophan (4.7 ± 0.1 ng (*E/Z*)-indole-3-acetaldoxime*h^−1^*assay^−1^) (Fig. 4b) suggests a higher turnover number for L-phenylalanine than for L-tryptophan.

### *Nicotiana benthamiana* expressing CYP79A61 produces phenylacetaldoxime, indole-3-acetaldoxime and phenylacetaldoxime-derived metabolites

**Fig. 5:**
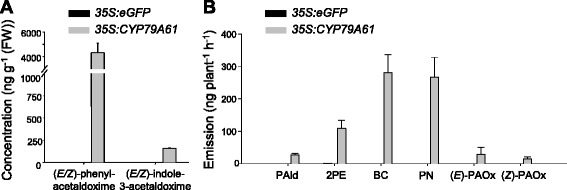
Aldoxime accumulation **a** and volatile emission **b** of transgenic *N. benthamiana* plants overexpressing maize *CYP79A61*. Plants were infiltrated with *A. tumefaciens* containing *35S:eGFP* (control) or *35S:CYP79A61.* Aldoximes were extracted three days after infiltration with methanol and analyzed using LC-MS/MS. Volatiles were collected on the third day after infiltration. Identification of volatile compounds was done with GC-MS and quantification was done with GC-FID. PAld, 2-phenylacetaldehyde; 2PE, 2-phenylethanol; BC, benzyl cyanide; PN, 2-phenylnitroethane; (*E*)-PAOx, (*E*)-phenylacetaldoxime; (*Z*)-PAOx, (*Z*)-phenylacetaldoxime. Means and standard errors are shown (*n* = 5)

To verify the biochemical properties of the recombinant protein in an *in vivo* plant system, *CYP79A61* was transferred into *Nicotiana benthamiana* using *Agrobacterium tumefaciens* and transiently expressed under control of the 35S promoter. As a negative control, a vector carrying the *35S::eGFP* fusion was used. A construct encoding the suppressor of silencing protein p19 [26] was coinfiltrated to increase transient protein expression. The *eGFP-*expressing plants showed a bright fluorescence on the 3rd day after infiltration. Thus, CYP79A61 products were analyzed 3 days after infiltration. To analyze potential volatile aldoxime products, a volatile collection was performed. Plants expressing the maize *CYP79A61* gene were found to release (*E*/*Z*)-phenylacetaldoxime in small amounts (Fig. 5b). In addition, some structurally related volatiles including 2-phenylacetaldehyde, 2-phenylethanol, benzyl cyanide, and 2-phenylnitroethane could be detected in the headspace of these plants (Fig. 5b, Additional file 1: Figure S4). In contrast, control plants expressing *eGFP* released none of the above-mentioned compounds. LC-MS/MS analysis of methanol extracts made from leaf material harvested right after the volatile collection revealed a strong accumulation of (*E/Z*)-phenylacetaldoxime and a moderate accumulation of (*E/Z*)-indole-3-acetaldoxime in leaves harboring the *35S::CYP79A61* construct, while no aldoximes could be detected in leaf material harvested from *eGFP-*expressing control plants (Fig. 5a).

### Caterpillar oral secretion induces *CYP79A61* gene expression as well as amino acid substrate accumulation and phenylacetaldoxime formation

**Fig. 6:**
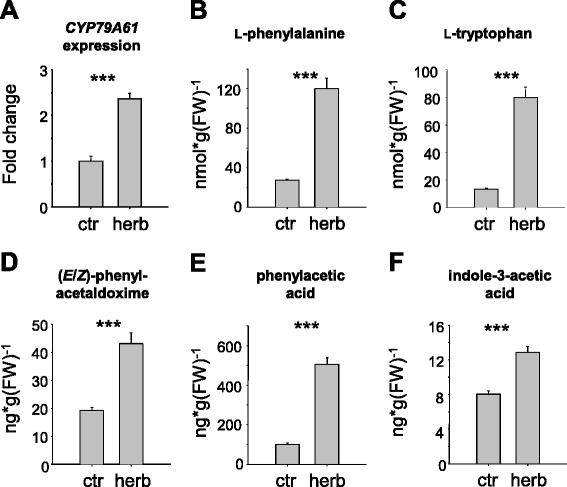
The response of maize leaves to simulated herbivory. *CYP79A61* gene expression **a**, L-phenylalanine **b** and L-tryptophan **c** accumulation, (*E/Z*)-phenylacetaldoxime content **d**, and phenylacetic acid **e** and indole-3-acetic acid **f** levels were measured in undamaged leaves (ctr) and leaves subjected to simulated herbivory (herb). (*E/Z*)-phenylacetaldoxime, L-phenylalanine, L-tryptophan, and the auxins phenylacetic acid and indole-3-acetic acid were extracted with methanol and analyzed by LC-MS/MS. Gene expression was determined by qRT-PCR. Means and standard errors are shown (*n* = 5). Asterisks indicate statistical significance in Student’s t-test. Gene expression: *p* < 0.001; *t* = −4.99; L-phenylalanine: *p* < 0.001, *t* = 15.242; L-tryptophan: *p* < 0.001, *t* = 16.293; phenylacetaldoxime: *p* = < 0.001, *t* = 6.934; phenylacetic acid: *p* = < 0.001 , *t* = −18.259; indole-3-acetic acid: *p* = < 0.001, *t* = −5.644

To test whether the expression of *CYP79A61* is influenced by herbivory, young maize plants of the cultivar Delprim were treated with oral secretion collected from Egyptian cotton leafworm (*Spodoptera littoralis*) larvae and *CYP79A61* transcript accumulation was analyzed in the leaves using quantitative (q)RT-PCR. While undamaged control plants showed a basal *CYP79A61* expression, simulated herbivory led to a significant increase in transcript accumulation (Fig. 6a). In contrast, *Spi1*, a member of the *YUCCA*-like gene family in maize which has been reported to be involved in indole-3-acetic acid formation [27], was not expressed in damaged and undamaged maize leaves (c_q_ values >39). LC-MS/MS analysis of L-phenylalanine and L-tryptophan in methanol extracts made from the same samples revealed a significant upregulation of both CYP79A61 substrates in response to the oral secretion treatment (Fig. 6b and c). (*E/Z*)-Phenylacetaldoxime showed a similar accumulation pattern with significantly higher amounts in damaged leaves than in undamaged controls (Fig. 6d). Indole-3-acetaldoxime, however, could not be detected in these leaf extracts.

### Caterpillar secretion induces the formation of the auxins indole-3-acetic acid and phenylacetic acid as potential aldoxime-derived metabolites

To investigate whether the maize cultivar Delprim is able to produce volatile aldoximes after herbivory, we conducted a volatile collection on plants treated with caterpillar oral secretions. Despite the accumulation of (*E*/*Z*)-phenylacetaldoxime in leaves, no aldoximes or aldoxime-derived nitriles or nitro compounds could be detected as volatiles (Additional file 1: Figure S5). However, several mono- and sesquiterpenes, green leaf volatiles and esters could be identified which have already been described in the literature [22, 23].

We then looked for potential metabolites of indole-3-acetaldoxime and phenylacetaldoxime since both are thought to be potential precursors for the biosynthesis of the auxins indole-3-acetic acid and phenylacetic acid (PAA), respectively [28], we searched for these metabolites in leaves of undamaged and oral secretion-treated maize plants. The accumulation of indole-3-acetic acid as well as the accumulation of phenylacetic acid was significantly increased in treated leaves in comparison to undamaged control leaves (Fig. 6e and f).

Since aldoximes are intermediates in the biosynthesis of cyanogenic glycosides, we also searched for these compounds in maize leaves. Maize has been reported as a cyanogenic plant species [17–19], but no cyanogenic glycosides have been identified so far. We used LC-MS/MS analysis to measure potential phenylacetaldoxime-derived cyanogenic glycosides, such as prunasin and amygdalin, as well as the *p*-hydroxyphenylacetaldoxime-derived cyanogenic glycoside dhurrin in oral secretion-treated maize leaves and in coleoptiles of maize and sorghum. As already reported in the literature [29, 30], dhurrin was found in large amounts in sorghum coleoptiles. However, none of the above mentioned cyanogenic glycosides could be detected in maize (Additional file 1: Figure S6), suggesting that at least the tested cultivar Delprim is not able to accumulate these compounds in significant amounts.

## Discussion

Aldoximes and aldoxime-derived compounds such as nitriles and cyanogenic glycosides are widespread secondary plant metabolites. They play important roles in plant defense against insects and pathogens [1, 3, 6, 11, 31] and are discussed to be involved in plant-pollinator interactions [32]. Although maize as one of the most important crop species has been intensively investigated during the last decades, little is known about the occurrence and role of aldoximes in this plant.

In this paper, we identified and characterized the P450 enzyme CYP79A61, one member of a small gene family comprising four genes with similarity to plant *CYP79*s. Like other CYP79 enzymes from the A- and B-subfamilies, recombinant CYP79A61 was shown to accept only aromatic amino acids as substrates. However, in contrast to most other CYP79 enzymes which have very high substrate specificity [5], both *in vitro* and *in vivo* experiments revealed that the recombinant maize enzyme was able to convert L-phenylalanine and L-tryptophan to phenylacetaldoxime and indole-3-acetaldoxime, respectively (Figs. 3 and 5). The conversion of a broader range of amino acids into aldoximes has only been reported for two poplar CYP79D enzymes [6]. The *K*_m_ values of CYP79A61 for L-phenylalanine and L-tryptophan were relatively high (*K*_m__(Phe)_ = 117.2 μM; *K*_m__(Trp)_ = 150.2 μM), but in the range reported for other CYP79 enzymes. It has been suggested that the low substrate affinity of these enzymes has evolved to avoid possible depletion of the free amino acid pool in plants [33].

The analysis of aldoximes in maize revealed a significant increase in phenylacetaldoxime accumulation in leaves treated with caterpillar oral secretion in comparison to leaves from undamaged control plants (Fig. 6d), suggesting a role of this compound in plant defense. Phenylacetaldoxime was previously shown to accumulate in poplar leaves after herbivory by gypsy moth (*Lymantria dispar*) caterpillars and feeding of pure phenylacetaldoxime to *L. dispar* larvae had negative effects on caterpillar survival, growth, and time until pupation [6]. Although the overall concentration of phenylacetaldoxime in maize leaves subjected to simulated herbivory (Fig. 6d) was relatively low compared to that found in poplar leaves, local formation of this compound giving higher concentrations around the wound site as already reported for defensive sesquiterpenes in maize [34] is conceivable. In addition, aldoximes have been suggested to play a role in plant defense against pathogens [10] and the accumulation of phenylacetaldoxime in treated maize leaves might thus represent a defense barrier against pathogen attack following insect herbivore damage. Apart from accumulating in plant tissue, aldoximes can serve as precursors for other defensive compounds [1–3, 35]. In the Japanese apricot (*Prunus mume*), for example, phenylacetaldoxime is converted into the cyanogenic glycosides prunasin and amygdalin [36]. This is unlikely to occur in maize since we could not detect these compounds neither in regurgitant-treated leaves nor in maize coleoptiles (Additional file 1: Figure S6), the developmental stage reported to possess the highest cyanogenic potential [19]. However, we cannot rule out that phenylacetaldoxime acts as a precursor for other so far unknown maize defense compounds.

Since CYP79A61 had similar *K*_m_ values for L-phenylalanine and L-tryptophan and both amino acids were found to accumulate in the same order of magnitude in maize leaves (Fig. 6b and c), one would expect that the enzyme produces equal amounts of phenylacetaldoxime and indole-3-acetaldoxime in planta. However, while phenylacetaldoxime was detected in maize leaves, no accumulation of indole-3-acetaldoxime could be observed (Fig. 6). Local differences in amino acid substrate concentrations caused, for example, by specific substrate channeling processes might be an explanation for this observation. However, it is far more likely that the lack of indole-3-acetaldoxime detection is due to the aldoxime being further converted into other compounds. In various plant species, including maize, the conversion of indole-3-acetaldoxime into the corresponding acid is thought to serve as an alternative route for the formation of the essential plant growth hormone indole-3-acetic acid [37–40], presumably involving indole-3-acetonitrile as an intermediate [37, 38]. The analysis of *CYP79A61* transcript accumulation in maize leaves revealed that the gene was significantly upregulated after herbivore feeding, matching an increased accumulation of IAA in the same tissues (Fig. 6a and f). Moreover, overexpression of *CYP79A61* in *N. benthamiana* revealed that the enzyme is able to produce indole-3-acetaldoxime under natural conditions in planta (Fig. 5a). Thus it is conceivable that CYP79A61 might produce indole-3-acetaldoxime as a specific substrate for herbivory-induced IAA formation in maize leaves. The conversion of indole-3-acetaldoxime to indole-3-acetonitrile is likely catalyzed by a P450 enzyme similar to the recently described poplar enzymes CYP71B40 and CYP71B41 which were shown to produce benzyl cyanide from phenylacetaldoxime after herbivory [35]. Indole-3-acetonitrile could then be further converted into IAA by maize nitrilase 2, an enzyme already implicated in auxin formation in maize [41]. In future experiments, the overexpression of maize *CYP79A61* in an Arabidopsis *cyp79b2 cyp79b3* double mutant which has been described to lack the accumulation of indole-3-acetaldoxime [40] would allow the analysis of *CYP79A61-*mediated formation of indole-3-acetaldoxime and its metabolism in a clean and sensitive background in planta. Since IAA can be formed via different biosynthetic pathways [28], it is possible that other enzymes rather than CYP79A61 are responsible for the observed IAA accumulation after simulated herbivory. Thus, a comprehensive expression analysis of candidate genes such as *TAA* and *YUCCA* might help to understand the biochemical origin of herbivore-induced IAA formation in maize. However, we have already shown that *Spi1*, a member of the YUCCA-like gene family in maize [27], was not expressed in damaged and undamaged maize leaves.

It is well established that herbivore feeding can cause changes in auxin levels in plants. For example, feeding of gall-inducing insects on wheat and late goldenrod (*Solidago altissima*) leads to increased IAA levels in the damaged tissues [42, 43] while simulated herbivory on wild tobacco (*Nicotiana attenuata*) resulted in decreased IAA accumulation [44]. Since auxins are potent modifiers of plant defense reactions [45], it is likely that the elevated IAA and PAA levels in herbivore-damaged maize also mediate defense responses. The presence of aldoxime-producing *CYP79* genes in all so far sequenced angiosperm genomes might indicate a broader occurrence of aldoxime-mediated auxin formation, especially under biotic stresses such as herbivory or pathogen attack.

A sequence comparison with already characterized CYP79s from other plants showed that CYP79A61 was most similar to CYP79A1, an enzyme known to catalyze the key reaction of dhurrin formation in sorghum [7]. However, despite an amino acid identity of 72 %, both enzymes have different substrate specificities with CYP79A1 solely converting tyrosine to *p*-hydroxyphenylacetaldoxime [46]. A comparative analysis of the maize and the sorghum genome revealed that *CYP79A61* and *CYP79A1* are not located on syntenic chromosomal regions and are therefore not orthologues (Additional file 1: Figure S1). Interestingly, no gene with orthology to sorghum *CYP79A1* could be found in the maize genome (Additional file 1: Figure S2), suggesting a recent loss of the *CYP79A1* orthologue in the maize lineage after diversification of the common ancestor of maize and sorghum. This gene loss might explain the absence of dhurrin formation in maize (Additional file 1: Figure S6). A so far uncharacterized sorghum *CYP79* gene (Sb10g022470) could be identified as the orthologue of *CYP79A61* (Additional file 1: Figures S2 and S3). However, whether this gene encodes for a protein with the same substrate specificity as CYP79A61 remains unknown.

Like dhurrin, we also could not detect the cyanogenic glycosides prunasin or amygdalin in the maize cultivar Delprim, neither in coleoptiles nor in undamaged or damaged leaves of young plants (Additional file 1: Figure S6). Moreover, a volatile collection experiment showed that Delprim did not release aldoximes after herbivory (Additional file 1: Figure S5). However, in the literature there is evidence that maize is cyanogenic [17–19], and a few maize lines have been reported to produce aliphatic volatile aldoximes after herbivore feeding [20, 21]. It is conceivable that the three putative *CYP79* genes GRMZM2G011156, GRMZM2G105185, and GRMZM2G178351, which could not be amplified from Delprim cDNA, are expressed in other maize cultivars or under different experimental conditions and contribute to volatile aldoxime and/or cyanogenic glycoside formation. Thus, a comprehensive characterization and gene expression analysis of different *CYP79* alleles from diverse maize cultivars will help to further understand the formation and function of these nitrogenous defense compounds and their variability among maize cultivars.

## Conclusions

We showed that maize produces aldoximes in response to simulated herbivory. A P450 enzyme of the CYP79 family, CYP79A61, could be identified able to catalyze the formation of phenylacetaldoxime and indole-3-acetaldoxime in two different heterologous systems. Since the expression of *CYP79A61* was upregulated after simulated herbivory, we hypothesize that the enzyme contributes to herbivore-induced aldoxime formation in maize. While phenylacetaldoxime accumulated in herbivore-damaged leaves and might play a role in maize defense against herbivores or pathogens, indole-3-acetaldoxime could not be detected in the plant. However, it is conceivable that this aldoxime is rapidly converted to indole-3-acetic acid which has been described as a mediator of various plant defense responses [45].

## Methods

### Plant and insect material

Seeds of the maize (*Zea mays* L.) hybrid line Delprim from Delley Samen und Pflanzen (Delley, Switzerland) were grown in commercially available potting soil in a climate-controlled chamber with a 16 h photoperiod (1 mmol (m^2^)^−1^ s^−1^ of photosynthetically-active radiation, temperature cycle 24/20 °C (day/night) and 60 % relative humidity). Twelve day old-plants (15–25 cm high, 4 expanded leaves) were used in the experiment. Eggs of *Spodoptera littoralis* Boisd. (Lepidoptera: Noctuidae) were obtained from Aventis (Frankfurt, Germany) and were reared on an artificial wheat germ diet (Heliothis mix, Stonefly Industries, Bryan, TX, USA) for about 10 days at 22 °C under an illumination of 750 μmol (m^2^)^−1^ s^−1^. Larvae were reared for another week on Delprim leaves and oral secretions were collected every day with a pipette and frozen at −20 °C until further usage. For the caterpillar secretion treatment (4 pm), 2 maize leaves per plant were cut with a razor blade and 15 μL oral secretion (1:2 diluted in water) were applied to the wound site. This treatment was repeated the next morning at 9 am prior to volatile collection.

### Volatile collection and analysis

For volatile collection, plants were separately placed in airtight 3 L glass desiccators. Charcoal-filtered air was pumped into the desiccators at a flow rate of 2 L min^−1^ and left the desiccators through a filter packed with 30 mg Porapaq Q (ARS, Inc., Gainesville, FL, USA). Volatiles were collected for 5 h (10 am – 3 pm). After collection the volatiles were desorbed by eluting the filter twice with 100 μL dichloromethane containing nonyl acetate as an internal standard (10 ng μL^−1^). Qualitative and quantitative analysis of maize volatiles was conducted using an Agilent 6890 Series gas chromatograph coupled to an Agilent 5973 quadrupole mass selective detector (interface temp.: 270 °C; quadrupole temp.: 150 °C, source temp.: 230 °C, electron energy: 70 eV) or a flame ionization detector (FID) operated at 300 °C, respectively. The constituents of the volatile bouquet were separated with a DB-5MS column (Agilent, Santa Clara, CA, USA, 30 m × 0.25 mm × 0.25 μm) and He (MS) or H_2_ (FID) as carrier gas. One microliters of the sample was injected without split at an initial oven temperature of 40 °C. The temperature was held for 2 min and then increased to 155 °C with a gradient of 7 °C min^−1^, followed by a further increase to 300 °C with 60 °C min^−1^ and a hold for 3 min.

Compounds were identified by comparison of retention times and mass spectra to those of authentic standards obtained from Fluka (Seelze, Germany), Roth (Karlsruhe, Germany), Sigma (St, Louis, MO, USA) or Bedoukian (Danbury, CT, USA), or by reference spectra in the Wiley and National Institute of Standards and Technology libraries and in the literature [47].

### Plant tissue sampling, RNA extraction and reverse transcription

Treated maize leaves were harvested immediately after the volatile collection (3 pm), flash-frozen in liquid nitrogen and stored at −80 °C until further processing. After grinding the frozen leaf material in liquid nitrogen to a fine powder, total RNA was isolated using the “RNeasy Plant Mini Kit” (Quiagen GmbH, Hilden, Germany) according to manufacturer’s instructions. RNA concentration, purity and quality were assessed using a spectrophotometer (NanoDrop 2000c, Thermo Scientific, Wilmington, DE, USA) and an Agilent 2100 Bioanalyzer (Agilent Technologies GmbH, Waldbronn, Germany). Prior to cDNA synthesis, 0.75 μg RNA was DNase-treated using 1 μL DNase (Fermentas GmbH, St. Leon Roth, Germany). Single-stranded cDNA was prepared from the DNase-treated RNA using SuperScript^TM^ III reverse transcriptase and oligo (dT_12–18_) primers (Invitrogen, Carlsbad, CA, USA).

### Identification and isolation of *CYP79* genes

To identify putative maize *CYP79* genes, a BLAST search against the *Z. maize* genome database (http://www.phytozome.net/poplar) was conducted using the amino acid sequence of CYP79A1 from *Sorghum bicolor* (L.) Moench (Genbank Q43135) as input sequence. Four sequences representing putative P450 enzymes of the CYP79 family were identified. One of these sequences could be amplified from cDNA attained from herbivore-induced leaves of *Z. mays.* Primer sequence information is available in Additional file 1: Table S1. The PCR product was cloned into the sequencing vector pCR®^−^Blunt II-TOPO® (Invitrogen) and both strands were fully sequenced.

### Heterologous expression of CYP79A61 in Saccharomyces cerevisiae

The complete open reading frame of *CYP79A61* was cloned into the pESC-Leu2d vector [24] as a *Not*I/*Bgl*II fragment and the resulting construct was transferred into the *S. cerevisiae* strain WAT11 [25]. For gene expression, a single yeast colony was picked to inoculate a starting culture which contained 30 mL SC minimal medium lacking leucine (6.7 g L^−1^ yeast nitrogen base without amino acids, but with ammonium sulfate). Other components: 100 mg L^−1^ of L-adenine, L-arginine, L-cysteine, L-lysine, L-threonine, L-tryptophan and uracil; 50 mg L^−1^ of the amino acids L-aspartic acid, L-histidine, L-isoleucine, L-methionine, L-phenylalanine, L-proline, L-serine, L-tyrosine, L-valine; 20 g L^−1^ D-glucose. The culture was grown overnight at 28 °C and 180 rpm. One OD of this culture (approx. 2 × 10^7^ cells mL^−1^) was used to inoculate 100 mL YPGA full medium (10 g L^−1^ yeast extract, 20 g L^−1^ bactopeptone, 74 mg L^−1^ adenine hemisulfate, 20 g L^−1^ D-glucose) which was grown for 32–35 h (until OD about 5), induced by the addition of galactose and cultured for another 15–18 h. Cells were harvested and yeast microsomes were isolated according to the procedures described by Pompon et al. [25] and Urban et al. [48] with minor modifications. Briefly, the culture was centrifuged (7500 *g*, 10 min, 4 °C), the supernatant was decanted, the pellet was resuspended in 30 mL TEK buffer (50 mM Tris-HCl pH 7.5, 1 mM EDTA, 100 mM KCl) and then centrifuged again. Then the cell pellet was carefully resuspended in 2 mL of TES buffer (50 mM Tris-HCl pH 7.5, 1 mM EDTA, 600 mM sorbitol, 10 g L^−1^ bovine serum fraction V protein and 1.5 mM β-mercaptoethanol) and transferred to a 50 mL conical tube. Glass beads (0.45–0.50 mm diameter, Sigma-Aldrich Chemicals, Steinheim, Germany) were added so that they filled the full volume of the cell suspension. Yeast cell walls were disrupted by 5 cycles of 1 min shaking by hand and subsequent cooling down on ice for 1 min. The crude extract was recovered by washing the glass beads 4 times with 5 mL TES. The combined washing fractions were centrifuged (7500 *g*, 10 min, 4 °C), and the supernatant was transferred into another tube and centrifuged again (100,000 *g* , 60 min, 4 °C). The resulting microsomal protein fraction was homogenized in 2 mL TEG buffer (50 mM Tris–HCl, 1 mM EDTA, 30 % *w/v* glycerol) using a glass homogenizer (Potter-Elvehjem, Fisher Scientific, Schwerte, Germany). Aliquots were stored at −20 °C and used for protein assays.

### Analysis of recombinant CYP79A61

To determine the substrate specificity of CYP79A61, yeast microsomes harboring recombinant protein were incubated for 30 min at 25 °C and 300 rpm individually with the potential substrates L-phenylalanine, L-valine, L-leucine, L-isoleucine, L-tyrosine and L-tryptophan in glass vials containing 300 μL of the reaction mixture (75 mM sodium phosphate buffer (pH 7.0), 1 mM substrate (concentration was variable for Km determination), 1 mM NADPH and 10 μL of the prepared microsomes). Reaction mixtures containing microsomes prepared from WAT11 transformed with the empty vector served as negative controls. Assays were stopped by placing on ice after 300 μL MeOH were added. Reaction products were analyzed using LC-MS/MS as described below. Product accumulation was measured after different incubation times (20–40 min) and under different pH conditions (pH 5.5–8.5). For the determination of the K_m_ values, assays were carried out as triplicates and enzyme concentrations and incubation times (30 min) were chosen so that the reaction velocity was linear during the incubation time period.

### qRT-PCR analysis of *CYP79A61* and *Spi1* expression

cDNA was prepared as described above and diluted 1:3 with water. For the amplification of the *CYP79A61* gene fragment (146 bp) and the *Spi1* gene fragment (99 bp), primer pairs were designed having a Tm ≥ 56 °C, a GC content between 52 and 56 % and a primer length in the range of 18–21 nt (see Additional file 1: Table S1 online for primer information). Primer specificity was confirmed by agarose gel electrophoresis, melting curve analysis, standard curve analysis, and sequence verification of cloned PCR amplicons. The transcription repressor Leunig (LUG) [49] was used as a reference gene. Samples were run in triplicates using Brilliant® III SYBR® Green QPCR Master Mix (Stratagene, Carlsbad, CA, USA) with ROX as reference dye. The following PCR conditions were applied for all reactions: Initial incubation at 95 °C for 3 min followed by 40 cycles of amplification (95 °C for 20 s, 60 °C for 20 s). Plate reads were taken during the annealing and the extension steps of each cycle. Data for the melting curves were recorded at the end of cycling from 55 to 95 °C.

All samples were run on the same PCR machine (Mx3000P, Agilent Technologies, Santa Clara, CA, USA) in an optical 96-well plate. Five biological replicates were analyzed as triplicates in the qRT-PCR for each of the three treatments. Data for the relative quantity to calibrator average (dRn) were exported from the MXPro Software.

### Transient expression of *CYP79A61* in *N. benthamiana*

For gene expression in *N. benthamiana*, the coding region of *CYP79A61* was cloned into the pCAMBiA2300U vector. After verification of the sequence integrity, pCAMBiA vectors carrying the *CYP79A61* or *eGFP* construct and the construct *pBIN::p19* were separately transferred into *Agrobacterium tumefaciens* strain LBA4404. The transformation was confirmed by PCR. Five milliliters of an overnight culture (220 rpm, 28 °C) were used to inoculate 50 mL LB medium (50 μg mL^−1^ kanamycin, 25 μg mL^−1^ rifampicin and 25 μg mL^−1^ gentamicin) for overnight growth. The following day, the cultures were centrifuged (4000 *g*, 5 min) and the cells were resuspended in infiltration buffer (10 mM MES, 10 mM MgCl_2_, 100 μM acetosyringone, pH 5.6) to reach a final OD of 0.5. After shaking for at least 1 h at RT, the cultures carrying *CYP79A61* or *eGFP* were mixed with an equal volume of cultures carrying *pBIN:p19*. Since p19 functions as a suppressor of silencing, it enhances the expression of the desired coexpressed protein in planta [26].

For transformation, 3–4 week-old *N. benthamiana* plants were dipped upside down in an *A. tumefaciens* solution and vacuum was applied to infiltrate the leaves. Infiltrated plants were shaded with cotton tissue to protect them from direct irradiation. Volatiles were measured on the 3rd day after transformation as described above.

### LC-MS/MS analysis of aldoximes, amino acids, auxins, and cyanogenic glycosides

For determining amino acid and aldoxime concentration, 100 mg of plant powder was extracted with 1 mL MeOH. For the measurement of amino acids, the MeOH extract was diluted 1:10 with water and spiked with ^13^C, ^15^ N abeled amino acids (algal amino acids ^13^C,^15^ N, Isotec, Miamisburg, OH, USA) at a concentration of 10 μg of the mix per mL. The concentration of the individual labeled amino acids in the mix had been previously determined by classical HPLC-fluorescence detection analysis after pre-column derivatization with *o*-phthaldialdehyde-mercaptoethanol using external standard curves made from standard mixtures (amino acid standard mix and Gln, Asn and Trp, Fluka). Amino acids in the diluted MeOH extract were directly analyzed by LC-MS/MS. The method described by Jander et al. [50] was used with some modifications. Briefly, chromatography was performed on an Agilent 1200 HPLC system (Agilent Technologies, Boeblingen, Germany). Separation was achieved on a Zorbax Eclipse XDB-C18 column (50 × 4.6 mm, 1.8 μm, Agilent Technologies) with aqueous formic acid (0.05 %) and acetonitrile employed as mobile phases A and B, respectively. The elution profile was: 0–1 min, 97 % A; 1–2.7 min, 3–100 % B in A; 2.7–3 min 100 % B and 3.1–6 min 97 % A. The mobile phase flow rate was 1.1 mL min^−1^ and the column temperature was maintained at 25 °C. The liquid chromatography was coupled to an API 5000 tandem mass spectrometer (Applied Biosystems, Darmstadt, Germany) equipped with a Turbospray ion source operated in positive ionization mode (ion spray voltage, 5500 eV; turbo gas temp, 700 °C; nebulizing gas, 70 psi; curtain gas, 35 psi; heating gas, 70 psi; collision gas, 2 psi). Multiple reaction monitoring (MRM) was used to monitor a parent ion → product ion reaction for each analyte. MRMs were chosen as described in Jander et al. [50] except for Arg (*m/z* 175 → 70), and Lys (*m/z* 147 → 84). Both Q1 and Q3 quadrupoles were maintained at unit resolution. Analyst 1.5 software (Applied Biosystems) was used for data acquisition and processing. Individual amino acids in the sample were quantified from corresponding peaks in the ^13^C,^15^ N labeled amino acid internal standard, except for tryptophan which was quantified using ^13^C,^15^ N-Phe applying a response factor of 0.42.

Aldoximes were measured from MeOH extracts as described in Irmisch et al. [6] using the same LC-MS/MS system as described above. Formic acid (0.2 %) in water and acetonitrile were employed as mobile phases A and B, respectively, on a Zorbax Eclipse XDB-C18 column (50 × 4.6 mm, 1.8 μm). The elution profile (gradient 1) was: 0–0.5 min, 30 % B; 0.5–3 min, 30–66 % B; 3–3.1 min, 66–100 % B; 3.1–4 min 100 % B and 4.1–6 min 30 % B at a flow rate of 0.8 mL min^−1^ at 25 °C. The API 5000 tandem mass spectrometer was operated in positive ionization mode (ion spray voltage, 5500 eV; turbo gas temp, 700 °C; nebulizing gas, 60 psi; curtain gas, 30 psi; heating gas, 50 psi; collision gas, 6 psi). MRM was used to monitor parent ion → product ion reactions for each analyte as follows: *m/z* 136.0 → 119.0 (collision energy (CE), 17 V; declustering potential (DP), 56 V) for phenylacetaldoxime; *m/z* 102.0 → 69.0 (CE, 13 V; DP, 31 V) for 2-methylbutyraldoxime; *m/z* 102.0 → 46.0 (CE, 15 V; DP, 31 V) for 3-methylbutyraldoxime; *m/z* 175.0 → 158.0 (CE, 17 V; DP, 56 V) for indole-3-acetaldoxime and *m/z* 152.0 → 107.0 (CE, 27 V; DP, 100 V) for *p*-hydroxyphenylacetaldoxime. The concentration of aldoximes was determined using external standard curves made with authentic standards synthesized as described in Irmisch et al. [6].

The auxins IAA and PAA were analyzed based on the protocol of Balcke et al. [51]. 100 mg of plant powder were extracted with 300 μL MeOH. Two hundred microliters of the extract was diluted 1:10 with water containing 0.1 % formic acid and loaded to equilibrated Chromabond® HR-X polypropylene columns (45 μm, Macherey Nagel, Düren, Germany). The columns were washed with acidified water. The fraction containing the auxins was eluted with 1 mL acetonitrile, which was then dried under a stream of nitrogen gas. The samples were redissolved in 30 μL MeOH and subsequently analyzed by the same LC-MS/MS system as described above. Separations were performed on an Agilent XDB-C18 column (50 × 4.6 mm, 1.8 μm). Eluents A and B were water containing 0.05 % formic acid and acetonitrile, respectively. The elution profile was: 0–0.5 min, 5 % B in A; 0.5–4.0 min, 5–50 % B; 4.1–4.5 min 100 % B and 4.6–7 min 5 % B. The flow rate was set to 1.1 mL min^−1^. For IAA analysis, the API 5000 tandem mass spectrometer was operated in positive ionization mode (ion spray voltage, 5500 eV; turbo gas temp, 700 °C; nebulizing gas, 60 psi; curtain gas, 30 psi; heating gas, 50 psi; collision gas, 6 psi). The MRM transition and parameter settings for IAA were as follows: *m/z* 176 → 130 (CE, 19 V; DP, 31 V). PAA was detected separately by mass spectrometer operated in negative ionization mode (ion spray voltage, −4500 eV; turbo gas temp, 700 °C; nebulizing gas, 60 psi; curtain gas, 30 psi; heating gas, 50 psi; collision gas, 6 psi). The MRM transition and parameter settings for PAA were as follows: *m/z* 135 → 91 (CE, −10 V; DP, −25 V). The concentration of PAA was determined using external standard curves made with authentic standard (Sigma-Aldrich). IAA concentration was determined internally by spiking the plant extracts with ^2^H_5_-IAA (OlChemIm Ltd., Olomouc, Czech Republic).

For the analysis of cyanogenic glycosides (dhurrin, prunasin, linamarin, and lotaustralin), 100 mg plant powder was extracted with 300 μL MeOH and 200 μL of the extract was diluted 1:10 with water containing 0.1 % formic acid. Ten microliters of the extracts were directly injected and analyzed by LC-MS/MS. The column and eluents used for the separation were the same as already described for the auxins. The elution profile was: 0–0.5 min, 5 % B in A; 0.5–6.0 min, 5–50 % B; 6.1–7.5 min 100 % B and 7.6–10.5 min 5 % B. The flow rate was set to 1.1 mL min^−1^. The tandem mass spectrometer was operated in negative ionization mode (ion spray voltage, −4500 eV; turbo gas temp, 700 °C; nebulizing gas, 60 psi; curtain gas, 30 psi; heating gas, 50 psi; collision gas, 6 psi). MRM was used to monitor parent ion → product ion reactions for each analyte as follows: *m/z* 310.0 → 179.0 for dhurrin, *m/z* 294.0 → 89.0 (CE, −22; DP, −15) for prunasin, *m/z* 260.0 → 179.0 for lotaustralin, *m/z* 246.0 → 179.0 for linamarin, and *m/z* 456.0 → 179.0 for amygdalin. If not stated above, the transition parameter settings for the cyanogenic glycosides were as follows: CE, −10 V; DP, −15 V.

### Sequence analysis and phylogenetic tree reconstruction

An alignment of maize CYP79 enzymes and CYP79A1 from *S. bicolor* was constructed and visualized using BioEdit (http://www.mbio.ncsu.edu/bioedit/bioedit.html) and the ClustalW algorithm. For the estimation of a phylogenetic tree, we used the ClustalW algorithm (gap open, 10; gap extend, 0.1; Gonnet; penalties, on; gap separation, 4; cut off, 30 %) implemented in MEGA5 [52] to compute an amino acid alignment of the maize CYP79 sequences and other already characterized CYP79 enzymes. The tree was reconstructed with MEGA5 using a neighbor-joining algorithm (Poisson model). A bootstrap resampling analysis with 1000 replicates was performed to evaluate the tree topology.

The synteny analysis was done using the EnsemblPlants web service (http://www.plants.ensembl.org).

### Statistical analysis

To test for statistical significance, data were log transformed whenever necessary and analyzed using the Student’s t-test implemented in SigmaPlot 11.0 for Windows (Systat Software Inc. 2008).

### Accession numbers

Sequence data for genes and proteins discussed in this article can be found in the GenBank under the following identifiers: CYP79A61 (KP297890), CYP79D6v3 (KF562515), CYP79D7v2 (KF562516), CYP79D3 (AAT11920), CYP79D4 (AAT11921), CYP79D1 (AAF27289), CYP79D2 (AAF27290), CYP79A1 (Q43135), CYP79B3 (AEC07294), CYP79B1 (AAD03415), CYP79B2 (AEE87143), CYP79A2 (AAF70255), CYP79E1 (AF140609), CYP79E2 (AF140610), CYP79F2 (AAG24796), CYP79F1 (AEE29448), CYP71E1 (AAC39318).

## Additional file

Additional file 1: Figure S1. Comparative genomic analysis of *Sorghum bicolor* chromosome 1 with maize chromosomes 1, 2, 5, and 9. The analysis was done using the web server http://www.plants.ensembl.org. **Figure S2.** Comparative genomic analysis of *Zea mays* chromosome 9 with *Sorghum bicolor* chromosomes 1 and 10. The analysis was done using the web server http://www.plants.ensembl.org. **Figure S3.** Phylogenetic tree of CYP79 sequences from maize and *Sorghum bicolor*. The rooted tree was inferred with the neighbor-joining method and *n* = 1000 replicates for bootstrapping. Bootstrap values are shown next to each node. As an outgroup, CYP71A13 from *Arabidopsis thaliana* was chosen. **Figure S4.** Volatiles released from transgenic *Nicotiana benthamiana* plants transiently overexpressing either a *35S::eGFP* construct or a *35S::CYP79A61* construct. Volatiles were collected 3 days after *Agrobacterium tumefaciens* infiltration and analyzed using GC-MS. 1, 5-*epi*-aristolochene; 2, 2-phenylethanol; 3, benzyl cyanide; 4, 2-phenylnitroethane; 5, phenylacetaldoxime; IS, internal standard. **Figure S5.** Volatiles released from undamaged 10 day-old *Zea mays* (cultivar Delprim) seedlings (control) and seedlings treated with caterpillar oral secretion (herbivory). Volatiles were collected and analyzed using GC-MS. 1, β-myrcene; 2, 3-hexen-1-ol acetate; 3, limonene; 4, linalool; 5, (*E*)-4,8-dimethyl-1,3,7-nonatriene; 6, phenylmethyl acetate; 7, 2-phenylethyl acetate; 8, indole; 9, geranyl acetate; 10, (*E*)-β-caryophyllene; 11, (*E*)-α-bergamotene; 12, (*E*)-β-farnesene; 13, β-sesquiphellandrene; 14, 4,8,12-trimethyltrideca-1,3,7,11-tetraene; IS, internal standard. **Figure S6.** Accumulation of cyanogenic glycosides in maize and sorghum. Maize and sorghum coleoptiles were harvested 3 days after germination. Undamaged maize leaves and caterpillar oral secretion-treated maize leaves were obtained as described in the Methods section. Glycosylated compounds were extracted with methanol and cyanogenic glycosides were analyzed using LC-MS/MS with multiple reaction monitoring (MRM). MRMs for dhurrin, prunasin, and amygdalin were established using authentic standards obtained from SIGMA-Aldrich (http://www.sigmaaldrich.com) (dhurrin) or prepared from bitter almonds (prunasin, amygdalin) and MRMs for lotaustralin and linamarin were calculated from those of dhurrin and prunasin. Amygdalin, lotaustralin and linamarin could not be detected in maize and sorghum (data not shown). **Table S1.** Oligonucleotides used in this study.

## Abbreviations

CYP: Cytochrome P450 monooxygenase
LC-MS/MS: Liquid chromatography-tandem mass spectrometry
IAA: Indole-3-acetic acid
PAA: Phenylacetic acid
FID: Flame ionization detector
MRM: Multiple reaction monitoring
