# Supplementary material for: The maize cytochrome P450 CYP79A61 produces phenylacetaldoxime and indole-3-acetaldoxime in heterologous systems and might contribute to plant defense and auxin formation
Source: BMC Plant Biol. 2015 May 29;15:128. doi: 10.1186/s12870-015-0526-1 (PMC4446944; doi:10.1186/s12870-015-0526-1)
Supplement: Additional file 1: Figure S1. — Comparative genomic analysis of Sorghum bicolor chromosome 1 with maize chromosomes 1, 2, 5, and 9. The analysis was done using the web server http://www.plants.ensembl.org. Figure S2. Comparative genomic analysis of Zea mays chromosome 9 with Sorghum bicolor chromosomes 1 and 10. The analysis was done using the web server http://www.plants.ensembl.org. Figure S3. Phylogenetic tree of CYP79 sequences from maize and Sorghum bicolor. The rooted tree was inferred with the neighbor-joining method and n = 1000 replicates for bootstrapping. Bootstrap values are shown next to each node. As an outgroup, CYP71A13 from Arabidopsis thaliana was chosen. Figure S4. Volatiles released from transgenic Nicotiana benthamiana plants transiently overexpressing either a 35S::eGFP construct or a 35S::CYP79A61 construct. Volatiles were collected 3 days after Agrobacterium tumefaciens infiltration and analyzed using GC-MS. 1, 5-epi-aristolochene; 2, 2-phenylethanol; 3, benzyl cyanide; 4, 2-phenylnitroethane; 5, phenylacetaldoxime; IS, internal standard. Figure S5. Volatiles released from undamaged 10 day-old Zea mays (cultivar Delprim) seedlings (control) and seedlings treated with caterpillar oral secretion (herbivory). Volatiles were collected and analyzed using GC-MS. 1, β-myrcene; 2, 3-hexen-1-ol acetate; 3, limonene; 4, linalool; 5, (E)-4,8-dimethyl-1,3,7-nonatriene; 6, phenylmethyl acetate; 7, 2-phenylethyl acetate; 8, indole; 9, geranyl acetate; 10, (E)-β-caryophyllene; 11, (E)-α-bergamotene; 12, (E)-β-farnesene; 13, β-sesquiphellandrene; 14, 4,8,12-trimethyltrideca-1,3,7,11-tetraene; IS, internal standard. Figure S6. Accumulation of cyanogenic glycosides in maize and sorghum. Maize and sorghum coleoptiles were harvested 3 days after germination. Undamaged maize leaves and caterpillar oral secretion-treated maize leaves were obtained as described in the Methods section. Glycosylated compounds were extracted with methanol and cyanogenic glycosides were analyzed using LC-MS/MS with m [file 12870_2015_526_MOESM1_ESM.pptx]

## Slide 1
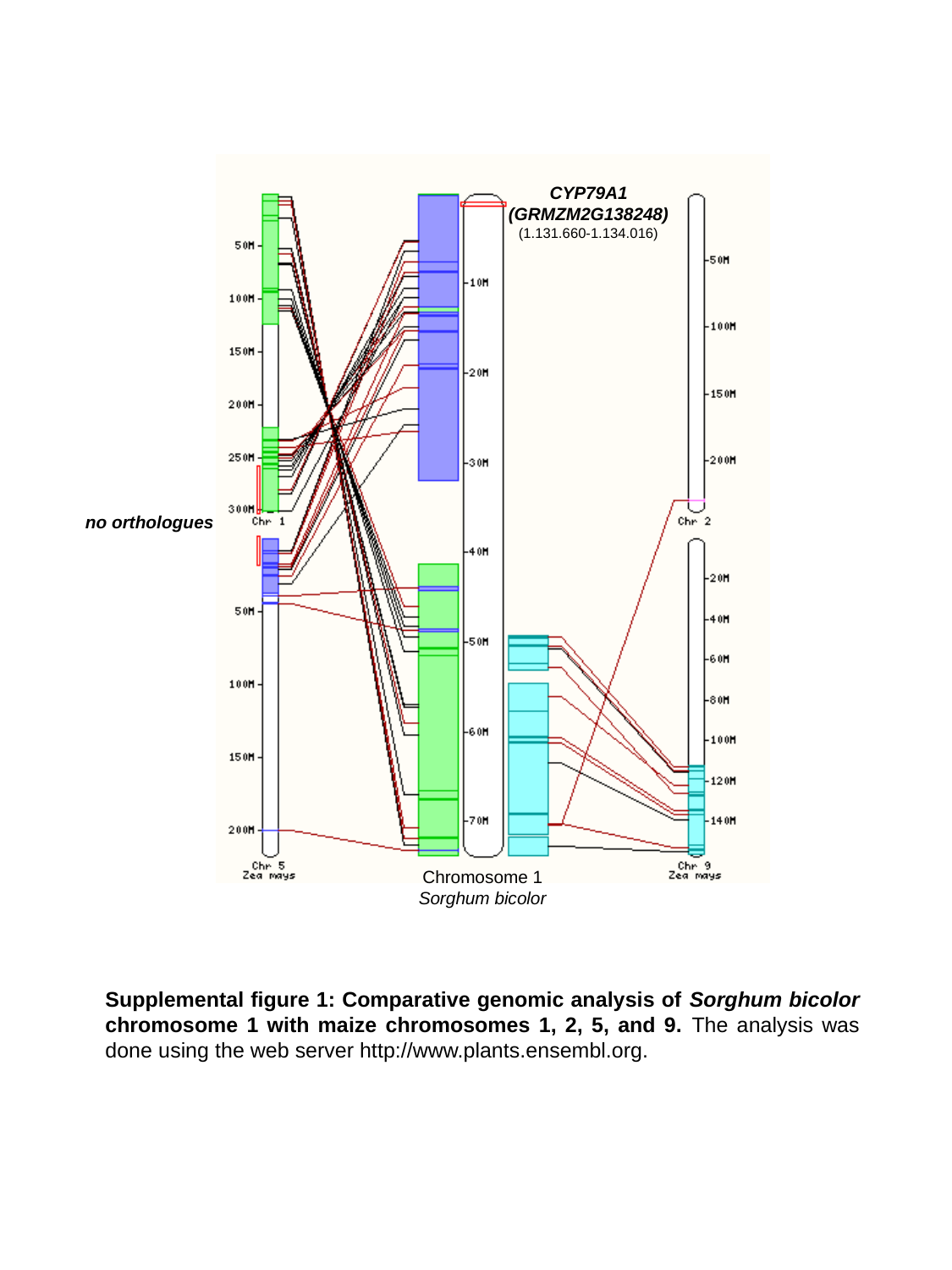

CYP79A1
(GRMZM2G138248)
(1.131.660-1.134.016)
no orthologues
Chromosome 1
Sorghum bicolor
Supplemental figure 1: Comparative genomic analysis of Sorghum bicolor chromosome 1 with maize chromosomes 1, 2, 5, and 9. The analysis was done using the web server http://www.plants.ensembl.org.

## Slide 2
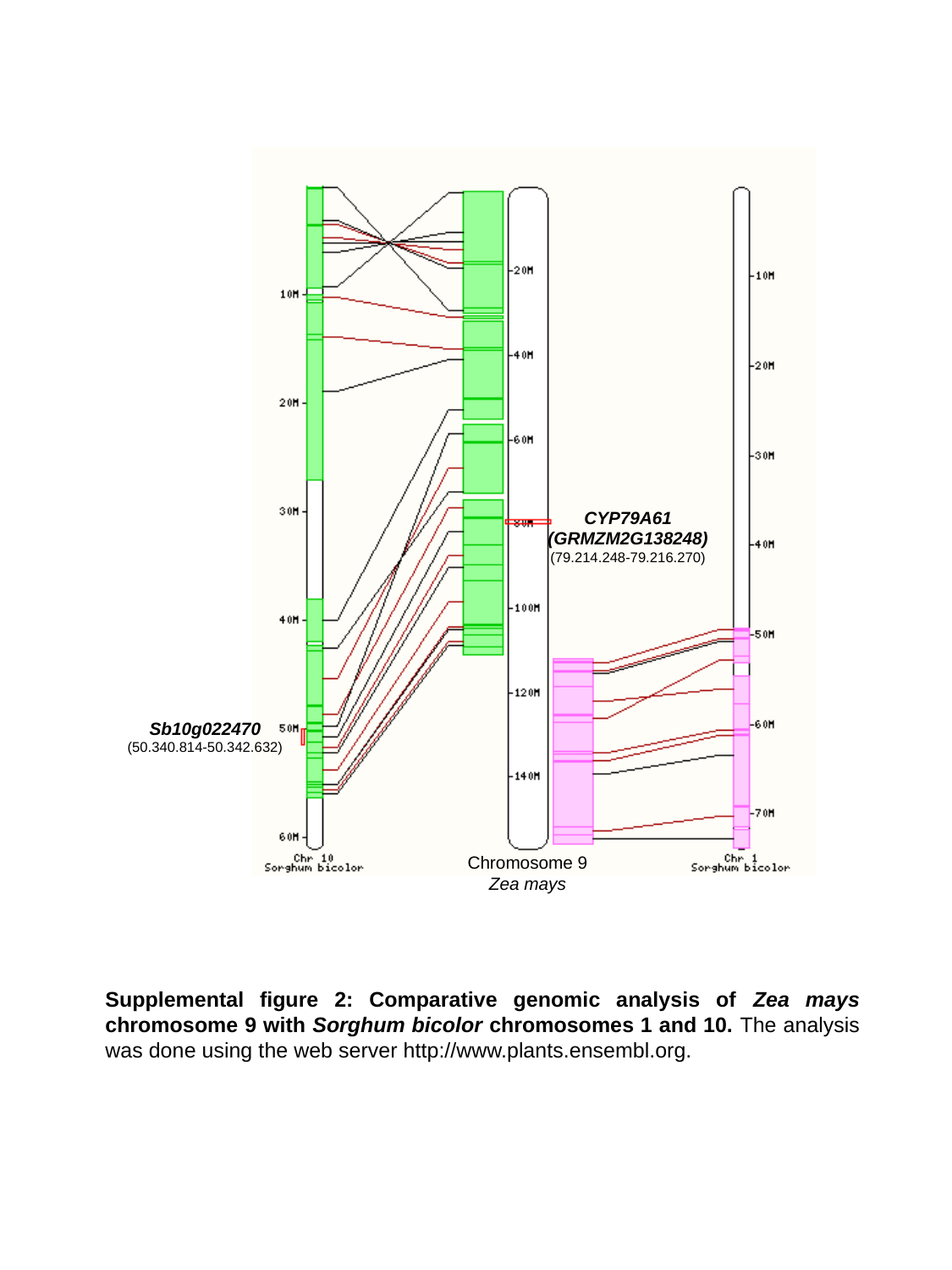

CYP79A61
(GRMZM2G138248)
(79.214.248-79.216.270)
Sb10g022470
(50.340.814-50.342.632)
Chromosome 9
Zea mays
Supplemental figure 2: Comparative genomic analysis of Zea mays chromosome 9 with Sorghum bicolor chromosomes 1 and 10. The analysis was done using the web server http://www.plants.ensembl.org.

## Slide 3
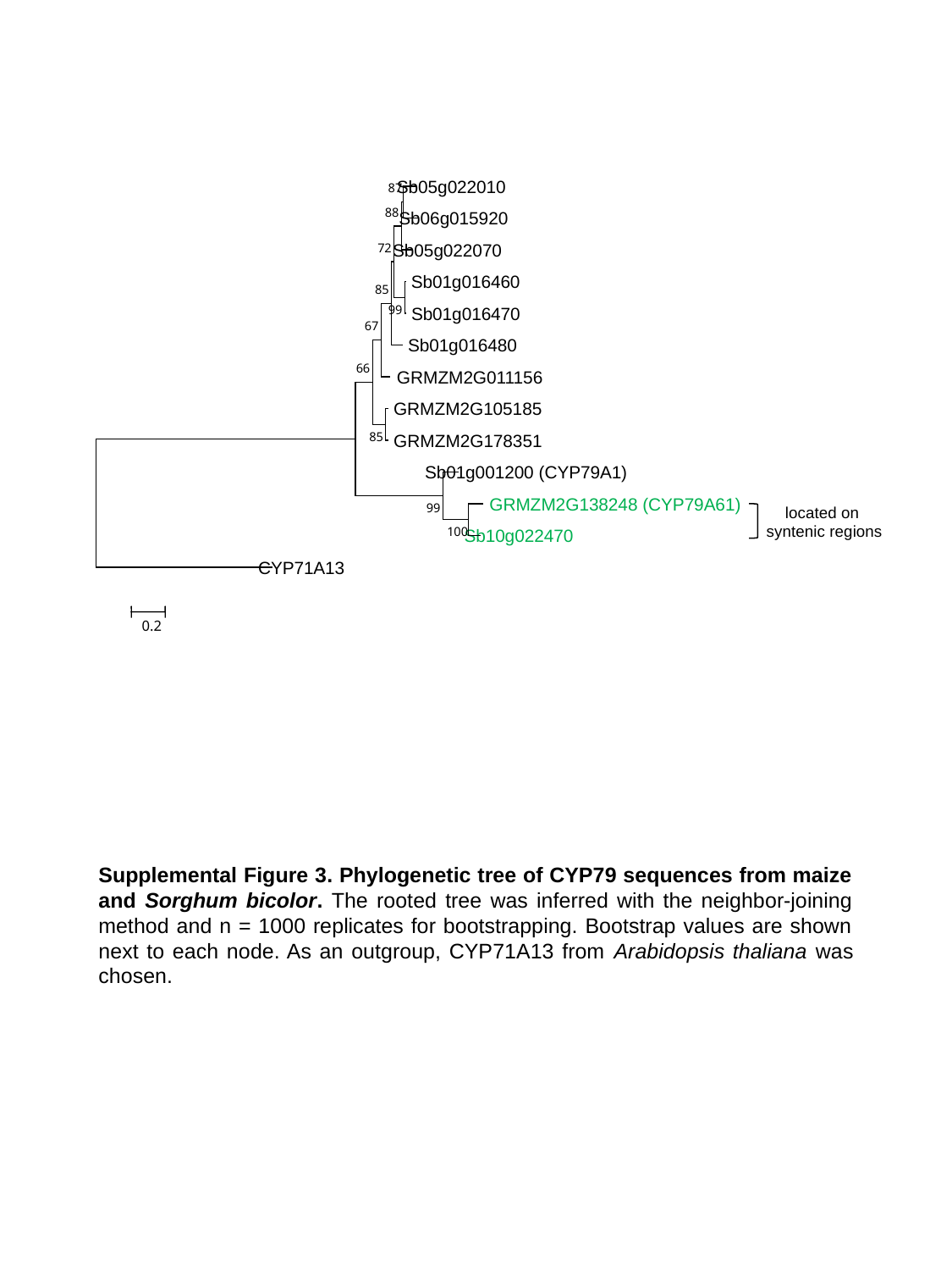

Sb05g022010
87
88
 Sb06g015920
 Sb05g022070
72
 Sb01g016460
85
99
 Sb01g016470
67
 Sb01g016480
66
 GRMZM2G011156
 GRMZM2G105185
85
 GRMZM2G178351
 Sb01g001200 (CYP79A1)
 GRMZM2G138248 (CYP79A61)
99
100
 Sb10g022470
 CYP71A13
0.2
located on
syntenic regions
Supplemental Figure 3. Phylogenetic tree of CYP79 sequences from maize and Sorghum bicolor. The rooted tree was inferred with the neighbor-joining method and n = 1000 replicates for bootstrapping. Bootstrap values are shown next to each node. As an outgroup, CYP71A13 from Arabidopsis thaliana was chosen.

## Slide 4
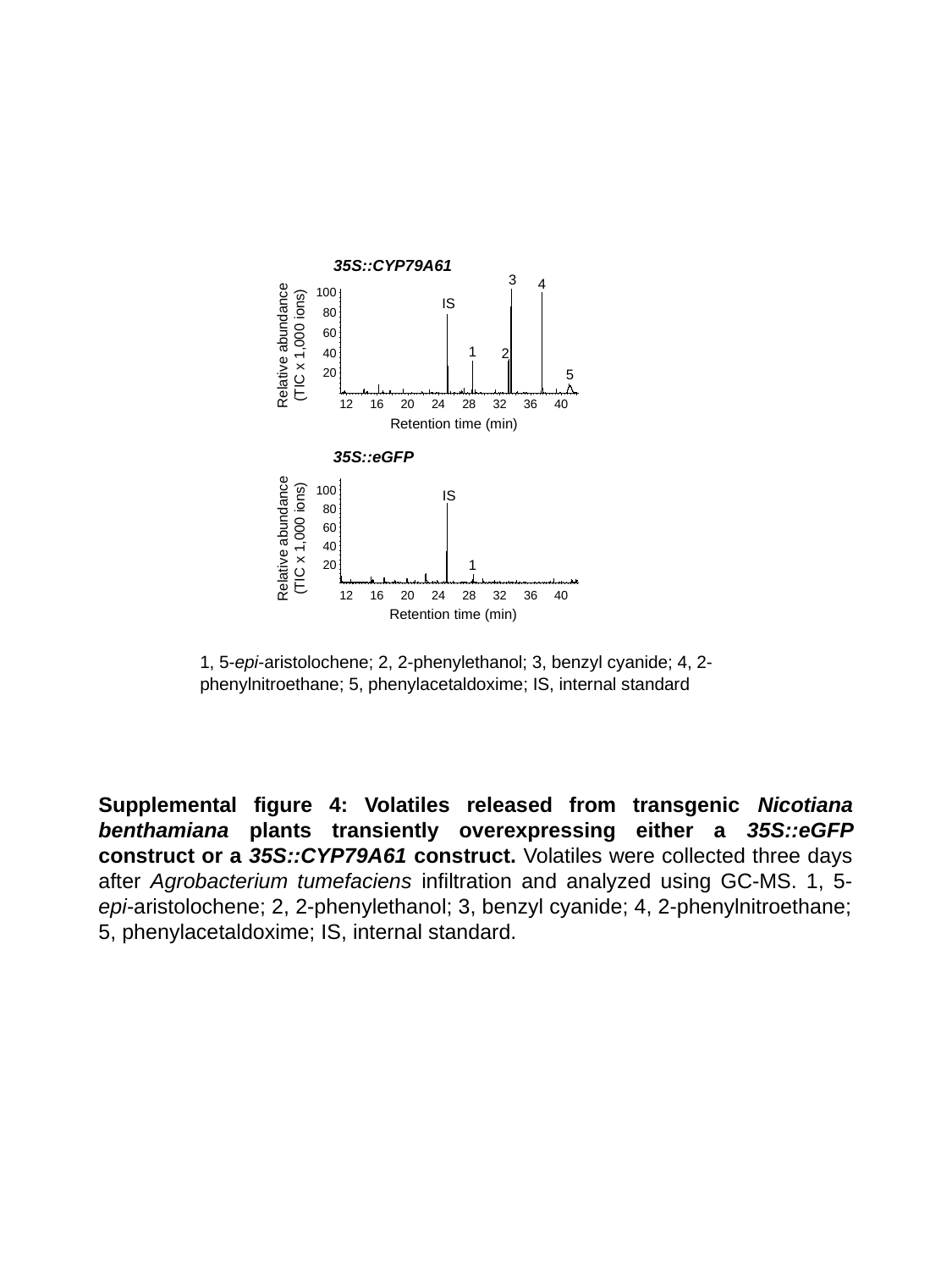

35S::CYP79A61
3
4
100
IS
80
Relative abundance
(TIC x 1,000 ions)
60
1
2
40
5
20
12
16
20
24
28
32
36
40
Retention time (min)
35S::eGFP
IS
100
80
Relative abundance
(TIC x 1,000 ions)
60
40
1
20
12
16
20
24
28
32
36
40
Retention time (min)
1, 5-epi-aristolochene; 2, 2-phenylethanol; 3, benzyl cyanide; 4, 2-phenylnitroethane; 5, phenylacetaldoxime; IS, internal standard
Supplemental figure 4: Volatiles released from transgenic Nicotiana benthamiana plants transiently overexpressing either a 35S::eGFP construct or a 35S::CYP79A61 construct. Volatiles were collected three days after Agrobacterium tumefaciens infiltration and analyzed using GC-MS. 1, 5-epi-aristolochene; 2, 2-phenylethanol; 3, benzyl cyanide; 4, 2-phenylnitroethane; 5, phenylacetaldoxime; IS, internal standard.

## Slide 5
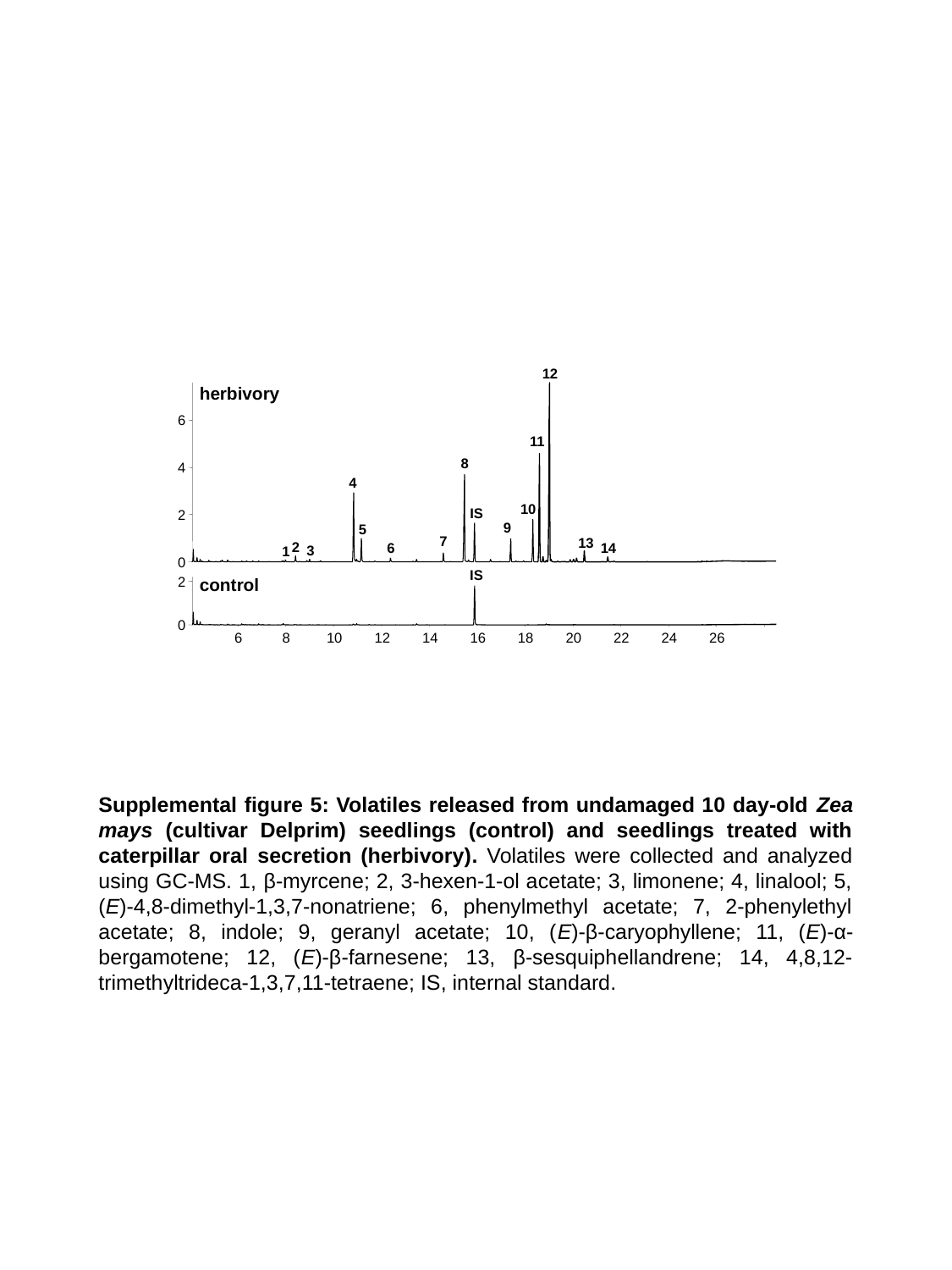

12
herbivory
6
11
8
4
4
10
IS
2
9
5
7
13
2
6
14
3
1
0
IS
2
control
0
6
8
10
12
14
16
18
20
22
24
26
Supplemental figure 5: Volatiles released from undamaged 10 day-old Zea mays (cultivar Delprim) seedlings (control) and seedlings treated with caterpillar oral secretion (herbivory). Volatiles were collected and analyzed using GC-MS. 1, β-myrcene; 2, 3-hexen-1-ol acetate; 3, limonene; 4, linalool; 5, (E)-4,8-dimethyl-1,3,7-nonatriene; 6, phenylmethyl acetate; 7, 2-phenylethyl acetate; 8, indole; 9, geranyl acetate; 10, (E)-β-caryophyllene; 11, (E)-α-bergamotene; 12, (E)-β-farnesene; 13, β-sesquiphellandrene; 14, 4,8,12-trimethyltrideca-1,3,7,11-tetraene; IS, internal standard.

## Slide 6
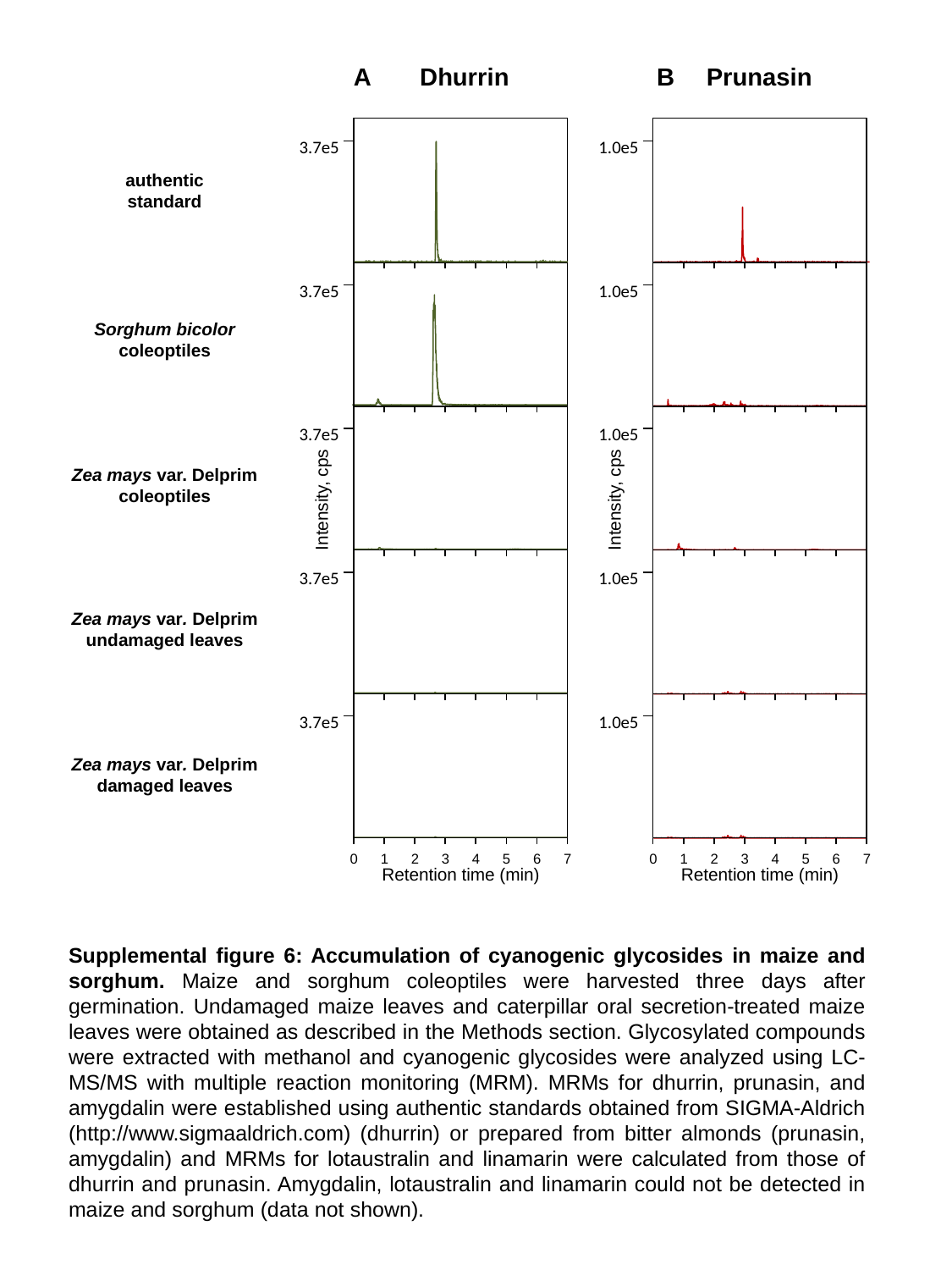

A
Dhurrin
B
Prunasin
3.7e5
3.7e5
3.7e5
3.7e5
3.7e5
1.0e5
1.0e5
1.0e5
1.0e5
1.0e5
authentic
standard
Sorghum bicolor
coleoptiles
Zea mays var. Delprim
coleoptiles
Intensity, cps
Intensity, cps
Zea mays var. Delprim
undamaged leaves
Zea mays var. Delprim
damaged leaves
0
1
2
3
4
5
6
7
0
1
2
3
4
5
6
7
Retention time (min)
Retention time (min)
Supplemental figure 6: Accumulation of cyanogenic glycosides in maize and sorghum. Maize and sorghum coleoptiles were harvested three days after germination. Undamaged maize leaves and caterpillar oral secretion-treated maize leaves were obtained as described in the Methods section. Glycosylated compounds were extracted with methanol and cyanogenic glycosides were analyzed using LC-MS/MS with multiple reaction monitoring (MRM). MRMs for dhurrin, prunasin, and amygdalin were established using authentic standards obtained from SIGMA-Aldrich (http://www.sigmaaldrich.com) (dhurrin) or prepared from bitter almonds (prunasin, amygdalin) and MRMs for lotaustralin and linamarin were calculated from those of dhurrin and prunasin. Amygdalin, lotaustralin and linamarin could not be detected in maize and sorghum (data not shown).

## Slide 7
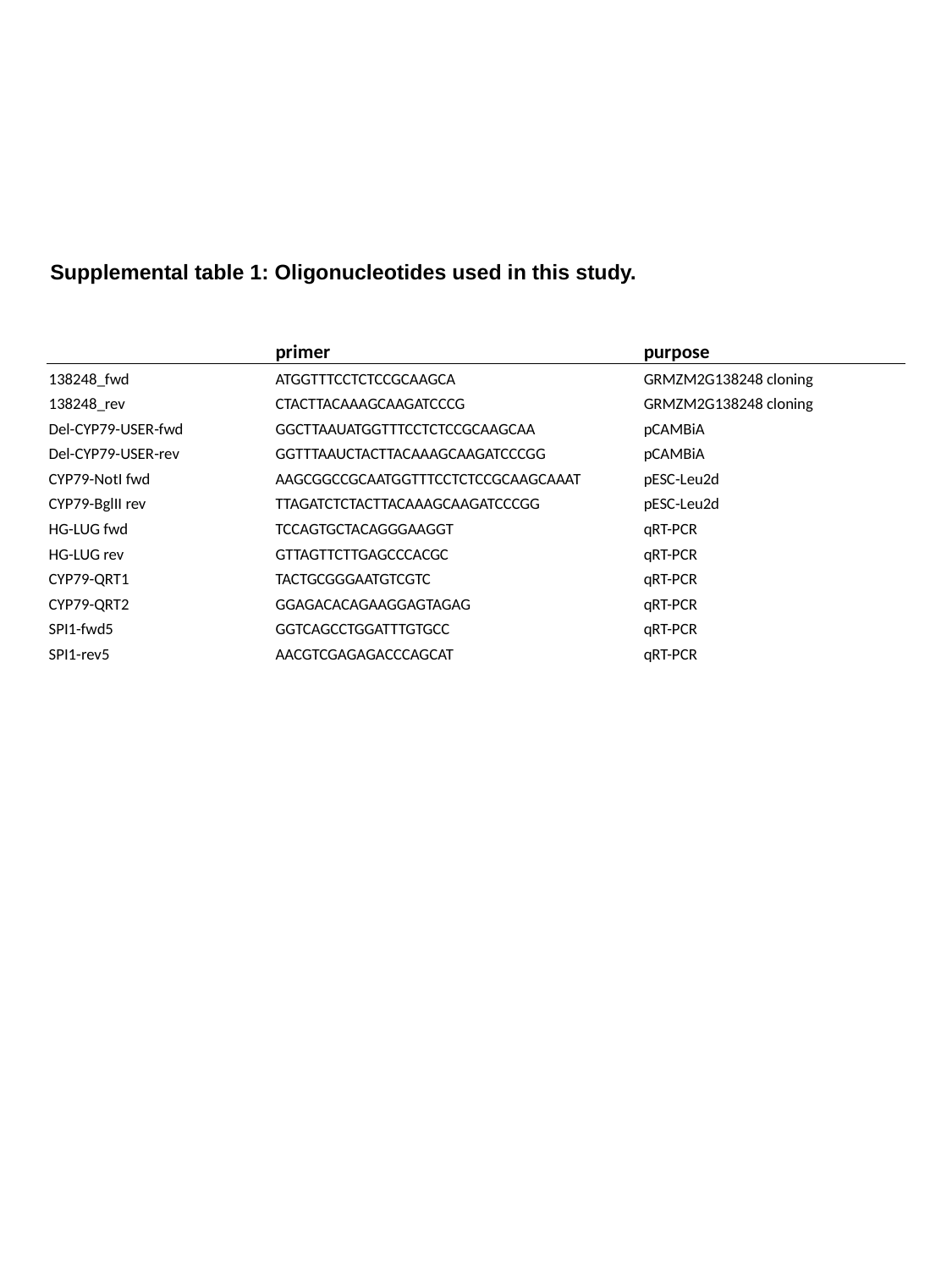

Supplemental table 1: Oligonucleotides used in this study.
| | primer | purpose |
| --- | --- | --- |
| 138248\_fwd | ATGGTTTCCTCTCCGCAAGCA | GRMZM2G138248 cloning |
| 138248\_rev | CTACTTACAAAGCAAGATCCCG | GRMZM2G138248 cloning |
| Del-CYP79-USER-fwd | GGCTTAAUATGGTTTCCTCTCCGCAAGCAA | pCAMBiA |
| Del-CYP79-USER-rev | GGTTTAAUCTACTTACAAAGCAAGATCCCGG | pCAMBiA |
| CYP79-NotI fwd | AAGCGGCCGCAATGGTTTCCTCTCCGCAAGCAAAT | pESC-Leu2d |
| CYP79-BglII rev | TTAGATCTCTACTTACAAAGCAAGATCCCGG | pESC-Leu2d |
| HG-LUG fwd | TCCAGTGCTACAGGGAAGGT | qRT-PCR |
| HG-LUG rev | GTTAGTTCTTGAGCCCACGC | qRT-PCR |
| CYP79-QRT1 | TACTGCGGGAATGTCGTC | qRT-PCR |
| CYP79-QRT2 | GGAGACACAGAAGGAGTAGAG | qRT-PCR |
| SPI1-fwd5 | GGTCAGCCTGGATTTGTGCC | qRT-PCR |
| SPI1-rev5 | AACGTCGAGAGACCCAGCAT | qRT-PCR |
